# Autistic Adults' Priorities for Future Autism Employment Research: Perspectives from the United Kingdom

**DOI:** 10.1089/aut.2022.0087

**Published:** 2024-02-28

**Authors:** Jade Davies, Anna Melissa Romualdez, Danae Malyan, Brett Heasman, Adam Livesey, Amy Walker, Elizabeth Pellicano, Anna Remington

**Affiliations:** ^1^ UCL Centre for Research in Autism and Education (CRAE), University College London, London, United Kingdom.; ^2^ School of Education, Language and Psychology, York St John University, York, United Kingdom.; ^3^ Neurodiversity Works, London, United Kingdom; ^4^ Clinical, Educational and Health Psychology, University College London, London, United Kingdom.; ^5^ Macquarie School of Education, Macquarie University, Sydney, Australia.

**Keywords:** employment, research priorities, priority-setting, participatory research

## Abstract

**Background:**

A growing body of research has sought to understand autistic people's research priorities. Several of these studies have identified employment as a key research priority. Yet, there have been a few attempts to identify specific, actionable priorities within this area.

**Methods:**

Using an online survey, we asked 197 autistic people in the United Kingdom about their priorities for future autism-employment research.

**Results:**

Participants spoke of their challenges in gaining and sustaining meaningful employment and called for researchers to conduct research that results in direct improvements to employment experiences. Regarding their research priorities, participants indicated a need for research covering all aspects of the employment lifecycle from accessing employment to transitioning out of employment. Importantly, participants also discussed *how* such research should be conducted: with autistic people as co-researchers and ensuring a diverse range of autistic people are listened to.

**Conclusion:**

While much existing autism-employment research appears to align with the priorities outlined in this study, seemingly minimal attention has been paid to later stages of the work lifecycle (e.g., progressing into more senior job roles or transitioning out of work). By identifying disparities between autistic people's priorities and the research being conducted, we can support autistic people to drive the research agenda and ensure autism-employment research positively impacts the community it aims to serve.

## Community brief

**Why was this study done?:**

It is important for autism research to identify and study topics that are important to autistic people. Employment has been highlighted as an important topic by autistic people in many studies. However, we don't know what specific topics employment research should look at.

**What was the purpose of this study?:**

We wanted to know which employment-related topics autistic people think are important to research and understand why.

**What did the researchers do?:**

We created an online survey to ask autistic people about what employment research should look at. The survey asked participants about themselves (e.g., their age) and their employment experience (e.g., if they were employed or not). We also asked questions about what employment topics should be researched, why these topics are important, and what changes employment research should lead to. In total, 197 autistic people responded. We read through all the responses and grouped similar responses together.

**What were the results of the study?:**

Lots of our participants found it hard to find and keep a job. They wanted research to make autistic peoples' experiences of employment better. For example, by making hiring processes better, and by making workplaces more inclusive. They also said that research should look at all parts of employment from getting a job, to leaving a job and retiring. Participants also talked about how research should be done. They said more autistic people should be included as researchers, and we should include different autistic people from different backgrounds.

**What do these findings add to what was already known?:**

Our findings show that lots of autism-employment research is on topics that autistic people think are important. However, not much research has looked at later stages of employment, such as getting a promotion or retiring.

**What are potential weaknesses in the study?:**

Most of our participants were White, female, employed and had a degree, but in the wider autistic population that isn't the case. People from other backgrounds may have different experiences of employment, and therefore want different research. We also don't know which topic is the most important because we didn't ask participants to rank the topics they spoke about.

**How will these findings help autistic adults now or in the future?:**

This study highlights key topics that autism-employment researchers should look at. By showing where the gaps in research are, we can make sure that all the employment topics autistic people think are important are researched.

## Introduction

Between the years 2001 and 2011, the number of annual autism research publications across the globe more than tripled,^1^ coinciding with worldwide increases in funding for autism research.^2,3^ For example, investment in autism research in the United Kingdom grew by 200% between the years 2013 and 2016, increasing from just £5 of research funding per autistic person in 2013 to £15 per person in 2016.^2^ Prompted by the exponential growth in research funding, recent work has sought to assess the distribution of research funding across topic areas.^1,2,4–7^ However, evidence suggests that the current allocation of autism research funding does not align with community research priorities.^8–11^ Such a disparity is concerning, risking frustration and a lack of trust in autism research from the community.^12^

To address this disparity, there have been calls for greater involvement of community members in the research process, including at the point of setting research priorities.^9,11,13–15^ Priority setting is common practice within health care research, with researchers establishing the common research needs and wants of the community they aim to serve and using these priorities to guide the direction of future research.^14^ Despite its potential utility, research concerning the autistic and autism communities' priorities for future autism research is in its infancy. Indeed, a recent systematic review of stakeholders' priorities for autism research identified only seven relevant academic publications, and only 9% of participants across those studies were autistic, meaning the priorities were set by mostly non-autistic people.^16^

In the few priority-setting exercises that have been conducted, employment research has consistently been prioritized by community members.^16^ For example, in Pellicano et al.'s study,^11^ autistic adults and parents of autistic children in the United Kingdom highlighted a need for research to establish “how to get [autistic] people into the workplace and keep them there” (p. 761). Similarly, when asked to rate the relative importance of 15 potential research topics (e.g., education, friendships, health care), autistic adults (*n* = 225) in the United States rated employment as the second highest research priority.^17^ Similar findings have also been replicated in Australia, with recent community consultations highlighting the development of inclusive workforces as a key “implementation research priority.”^18–20^

The prioritization of employment research is perhaps unsurprising given that employment forms a significant part of most people's daily life. Indeed, employment provides important opportunities for financial independence, and developing and maintaining a sense of purpose.^21^ Further, employment has important implications for autistic people's social experiences, as well as their overall quality of life.^22,23^ Notwithstanding, a significant proportion of autistic people across the globe are unemployed.

For example, fewer than 22% of autistic adults in the United Kingdom are employed, with similar statistics being reported in Canada (14.3% employed) and Australia (28% employed).^24–26^ Consequently, many autistic people and their families are left with growing concerns regarding their current and future physical and mental wellbeing, financial security, and independence.^27–30^

Autistic people that *have* secured employment also report facing significant challenges, including (1) deciding whether to disclose their diagnosis to employers^31–33^ (see Lindsay et al.^34^ for an earlier review); (2) accessing workplace adjustments^35^ (see Khalifa et al.^36^ and Lindsay et al.^34^ for previous reviews); and (3) facing significant stigma and discrimination.^37–39^ Similarly, many employed autistic people face underemployment (e.g., are over-qualified for the role they are in) and are, therefore, not given the opportunity to reach their potential.^40–42^

Despite the clear need and desire for autism employment research, there is a paucity of research designed to establish the specific employment-related topics that are prioritized by autistic people. Two studies from the United States (US) have attempted to identify priorities in relation to the *transition* into employment.^43,44^ However, entering employment is only one aspect of the employment journey, and the priorities of autistic adults who may already have employment experience are likely to differ from the priorities of those entering employment for the first time.

In one of the only consultations to identify broader employment-related research priorities, the Australian Autism Research Council^18^ held online focus groups with autistic people and their allies (family members, and/or professionals that work with autistic people) to identify key research questions in five community-informed priority areas: education, health and well-being, employment, justice, and communication. Regarding employment specifically, the project team asked a small number of “experts” (*n* = 12, 9 of whom were autistic) to engage in a prolonged discussion about their priorities for autism-employment research. Participants were then encouraged to identify a set of employment-specific research questions, resulting in a list of 10 key priority questions for future autism-employment research.^18^ The top three employment-related research priorities highlighted in this consultation included (a) understanding how the needs and preferences of autistic people can be supported in the workplace (e.g., through workplace adjustments); (b) identifying the factors that create a safe work environment and culture for autistic employees; and (c) improving recruitment processes for autistic people.^18^

While the earlier cited consultation was useful in identifying potential priority areas for future autism-employment research, what is lacking is an in-depth consultation with a large sample of autistic people, outside the Australian employment context. In this study, we conducted a large-scale online survey with autistic adults in the United Kingdom. Specifically, we wanted to understand (a) what autistic people think future autism-employment research should cover; (b) what autistic people think future autism-employment research should seek to achieve; and (c) *why* these research topics and outcomes are important to address. Attending to the latter aim is critically important in both contextualizing the identified priorities and understanding how research practices may improve going forward.

## Methods

This study formed part of a larger research initiative, Discover Autism Research and Employment (DARE). DARE aims to explore autistic adults' experiences of employment in the United Kingdom using a bespoke national survey, called the Diverse Minds Survey, developed with autistic collaborators. The Diverse Minds Survey includes optional modules on seven employment-related topics, including priorities for future autism-employment research.

We advertised the survey via (a) social-media callouts by individual members of the research team; (b) advertisements through the Autistica Discover Network, a network of autistic people interested in taking part in research; and (c) callouts within organizations linked to the project that were interested in learning more about neurodiversity and employment.

### Participants

To take part in the Diverse Minds Survey, participants were required to be aged 18 years or older and have experience of employment, or searching for employment, in the United Kingdom. To be included in the current study, participants also had to complete all demographic questions and at least one question from the priorities for future employment research module. We excluded participants who did not identify as autistic.

Between March 2019 and April 2020, 347 people had navigated to the priorities for future employment research survey. Of those, we excluded 150 (43.2%) because they did not identify as autistic (*n* = 145, 96.7%), or had not answered any of the relevant research priorities questions (*n* = 5, 3.3%). The final sample comprised 197 participants. Of those, the majority reported having received a formal autism diagnosis (*n* = 168, 85.3%), with the remaining self-identifying as autistic (*n* = 28, 14.2%). One participant (0.5%) stated they were autistic but did not disclose whether they had a formal diagnosis.

More than half of the sample (*n* = 113, 57.4%) identified as female and more than three-quarters (*n* = 153, 77.7%) were from a White ethnic background. Many participants* were in full-time (*n* = 72, 36.5%) or part-time (*n* = 42, 21.3%) employment, or they were self-employed (*n* = 16, 8.1%). Less than half of the sample (*n* = 86, 43.7%) reported being satisfied with their current employment. See Table 1 for a full breakdown of demographic information.

**Table 1.** Participant Demographic Information (*n* = 197)

	*n*	%
Gender		
Female (including trans women)	113	57.4
Male (including trans men)	65	33.0
Non-binary	13	6.6
Prefer to self-describe	6	3.0
Age category (in years)		
18–25	28	14.2
26–35	44	22.3
36–45	44	22.3
46–55	58	29.4
56–65	21	10.7
66–75	2	1.0
Ethnicity		
White	153	77.7
Mixed/multiple ethnic backgrounds	10	5.1
Black British	1	0.5
Undisclosed	6	3.0
Undetermined^a^	27	13.7
Highest level of education		
Doctorate	13	6.6
Master's Degree (e.g., MA, MSc, MEd)	54	27.4
Post Graduate Certificate (PGCert)	8	4.1
Post Graduate Diploma (PGDip)	6	3.0
Bachelor's Degree (e.g., BSc, BA, BEd)	57	28.9
Higher National Diploma (HND)^b^	5	2.5
Foundation Degree	5	2.5
A/AS-Level^c^	17	8.6
GCSEs^d^	6	3.0
BTEC/GNVQ^e^	16	8.1
No formal qualifications	5	2.5
Other	5	2.5
Employment status		
Employed full-time	72	36.5
Employed part-time	42	21.3
Self-employed	16	8.1
Unemployed—looking for work	13	6.6
Student	12	6.1
Unemployed—not looking for work	12	6.1
Volunteer	6	3.0
Parent and/or carer	5	2.5
Unemployed—disabled/ill-health	5	2.5
Retired	4	2.0
Other	10	5.1
Satisfaction with current employment		
Satisfied	86	43.7
Dissatisfied	55	27.9
Uncertain	45	22.8
Not applicable	4	2.0
Other	7	3.6
Number of previous employers		
None	5	2.5
1–2 Employers	21	10.7
2–4 Employers	42	21.3
4–6 Employers	38	19.3
>6 Employers	88	44.7
Prefer not to say	3	1.5
Income range		
<£10,000	45	22.8
£10,000–£19,999	48	24.4
£20,000–£29,999	39	19.8
£30,000–£39,999	23	11.7
£40,000–£49,999	9	4.6
£50,000–£59,999	5	2.5
£60,000–£79,999	7	3.6
£80,000–£99,999	3	1.5
£100,000–£149,999	4	2.0
Prefer not to say	14	7.1
Top 5 most common employment sectors		
Education	30	15.2
Health care	20	10.2
Public sector	17	8.6
Administration	14	7.1
IT^f^	11	5.6

^a^ Participants were given a free text response option for ethnicity. Some participants chose to report their nationality as opposed to their ethnicity. These responses have been categorized as undetermined.

^b^ A HND is equivalent to the second year of a Bachelor's degree.

^c^ AS/A-Levels are qualifications in the United Kingdom that are typically taken between 16 and 18 years.

^d^ GCSEs are qualifications in the United Kingdom that are typically taken between 14 and 16 years.

^e^ BTEC and GNVQ qualifications are specialist work-related qualifications in the United Kingdom that start at GCSE-equivalent level. GNVQs were replaced by BTECs in 2007.

^f^ IT, information technology.

## Materials

Participants completed a series of demographic questions regarding their age category, gender identity, ethnicity, highest level of education, employment status, satisfaction with current employment, number of previous employers, income range, and sector of employment. Participants also completed an optional module regarding their priorities for future employment research. The priorities for future employment research module consisted of short answer responses to three open-ended questions regarding (a) the areas of autism and employment they would like to see researched in the future; (b) existing autism-employment topics that should be researched from another angle; and (c) in what ways they would like to see future employment research make a difference to the employment of autistic people.

### Procedure

We obtained ethical approval through the Research Ethics Committee at University College London (UCL) Institute of Education, UCL's Faculty of Education and Society (REC1149). Participants provided informed consent to take part in the research. The priorities for future research module took ∼10 minutes to complete.

### Data analysis

We uploaded survey responses (average length = 46 words, range = 2–420 words) to NVivo software and analyzed them using reflexive thematic analysis, within an essentialist framework.^45,46^ We chose to use reflexive thematic analysis, because we wanted to identify research priorities within the context of individual experiences (i.e., to both identify priority research topics and understand *why* these topics were prioritized). The first author led the data analytic process, familiarizing themselves with the data by reading and rereading responses to the three open-ended questions.

We adopted an inductive approach (i.e., without integrating the themes within any pre-existing coding schemes or researcher preconceptions) to identify patterned meanings within the dataset. We assigned initial codes to data items that were perceived to be useful in addressing the research questions, taking an inductive approach. We utilized both semantic and latent coding, recursively reviewing and refining codes. Next, similar and/or complementary codes were grouped, and initial themes and sub-themes were generated. The first author met with the second author, who independently read a subset of the data, on multiple occasions to discuss their interpretations of the data and develop a richer understanding of the underlying meaning. All authors read through, and agreed upon, the final set of themes and sub-themes.

Regarding positionality, all authors are part of the autism community, either as a self-advocate (A.L. and A.W.), or as an ally and/or researcher (J.D., A.M.R., D.M., B.H., E.P., and A.R.). The authors all view autism within a neurodiversity framework, and recognize that disability is, at least in part, a consequence of societal barriers that exclude or discriminate against autistic people, rather than inherent impairments or deficits. While all authors share this view, they nevertheless have different levels of experience and expertise (e.g., lived-experience, junior research experience, senior research experience) within autism research and therefore contributed unique insights and interpretations to the data. The themes and subthemes were reflexively refined considering researcher biases and were sense-checked through intense discussion with the third author, who was familiar with the dataset.^47^

### Community involvement

Two autistic co-authors (A.L. and A.W.) contributed to the development of the Diverse Minds Survey. This included identifying key topics, designing survey questions, and refining the survey to improve accessibility, such as adjusting the format of the survey and the language used. A.L. and A.W. also provided crucial feedback on drafts of the manuscript, including key recommendations for future research.

## Results

### Qualitative results

**FIG. 1.**
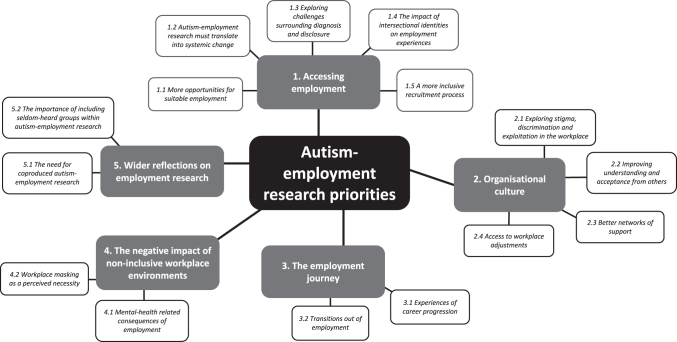
Thematic map of autistic people's priorities for future autism-employment research.

We identified 5 themes comprising 15 subthemes from the free-text responses (Fig. 1). Themes focused on accessing employment (Theme 1); organizational culture (Theme 2); the employment journey (Theme 3); the negative impact of non-inclusive workplace environments (Theme 4); and wider reflections on autism-employment research (Theme 5). The research team developed example research questions under each theme and sub-theme, directly drawn from participants' examples and anecdotes. Readers can find example research questions in Supplementary Appendix Table SA1.

#### Theme 1: accessing employment

##### More opportunities for suitable employment

Participants discussed a need to understand “the reasons for the employment gap” (P090) to “increase the numbers of autistics in employment” (P048). One possible reason was a perceived lack of opportunities for suitable and meaningful employment for autistic people. Indeed, some participants felt they had previously been “written off as only suitable for some minimum wage job” (P008). As such, many participants reported being in unfulfilling job roles that did not adequately match their capabilities or utilise their skills.

Relatedly, one participant emphasized a need for researchers to move their focus from simply increasing employment rates to supporting autistic people to access more meaningful employment: “I get it, there's a need to find more jobs for people with autism, but when is there going to be an emphasis on *careers* for people with autism?” (P103). As a result of the limited suitable opportunities, some participants felt they had no choice but to turn to self-employment: “the workplace is not designed for neurodivergent people. I am extremely successful in my work … but I am self-employed as I do not fit into a workplace” (P029).

Participants offered several suggestions for how research could support more autistic people to access suitable employment, including research highlighting the “types of jobs that suit autistics” (P044), the “fields which are more autism friendly than others” (P152) and “employers [that] actively recruit autistic people” (P144). Some participants also felt that they would benefit from research on external forms of support, such as “mentoring which employed autistic adults might give to autistic school leavers about to enter the workplace” (P018).

##### Autism-employment research must translate into systemic change

Participants wanted wider, systemic changes such as “governmental understanding” (P118) and “government backed schemes” (P144) to make a real improvement to the accessibility of employment for autistic people. They emphasized that research must result in real, tangible change, for example, by resulting in “a [national] autism employment fund to help fund work experience, internships, and job opportunities for autistic people” (P148), or by resulting in direct improvements to current UK laws, and the benefit and welfare system: “[the] current law needs updating as it is a sham right now, it only means employers have a duty to meet the needs as a disabled group, not individuals” (P044).

##### Exploring challenges surrounding diagnosis and disclosure

Participants also felt it was important for research to examine the implications of diagnostic disclosure to access reasonable adjustments. For example, some participants highlighted that there are likely many autistic employees “working without a diagnosis … [and] employers will very likely be employing people who are autistic but don't know it, so don't know even to ask for adjustments” (P171). Indeed, participants noted that the lack of adjustments for people without an autism diagnosis often led to unique “difficulties in securing or retaining paid employment” (P132). As such, participants suggested that research should examine “how [autistic people] can best get what they need without having to disclose personal information to an employer that [they] don't fully trust” (P050).

For some autistic people, however, disclosure was an important goal: “I would like people to feel safe and comfortable disclosing” (P053). Research targeted to this issue was, therefore, perceived to be an important catalyst for change, potentially providing autistic people with “different ways of disclosing autism in the workplace” (P163) as well as making “it normal for people with autism to be able to disclose their diagnosis from the outset, just like someone might say that English is their second language, or that they are left-handed” (P088).

##### The impact of intersectional identities on employment experiences

Some participants were particularly interested in understanding how having an intersectional identity may exacerbate some of the barriers that autistic people face in accessing employment. For example, one participant explained: “I want [researchers] to examine how more than one ‘persona-trait’ (e.g., being autistic *and* being a woman, *and*/or being of non-White ethnicity, *and*/or being physically disabled) [may] impact even more negatively on judgements in employment situations” (P137). Indeed, participants shared anecdotes of subgroups in the autistic community that they perceived as doubly disadvantaged because of their intersectional identity:

[Autistic people] from middle class families are supported to achieve an education, often through home-schooling. Those from working class backgrounds but of similar intelligence, often end up as “school refusers” and reach 18 without a single qualification. This obviously has a big impact on employment. My sense is there is appalling inequality based on family class and resources. It would be interesting to see this researched. (P008)

##### A more inclusive recruitment process

Typical recruitment processes, including interviews and group tasks, were thought to be “the most significant barrier [to employment] for many neurodivergent individuals” (P188). Participants reported that “more [autistic people] could be employed if recruitment processes and workplaces were more autistic friendly” (P173). As such, participants felt it was important for research to highlight “autism friendly recruitment best practice” (P171) and indicate “how application [and] recruitment processes could be made easier and more practical for people with autism” (P093). Participants hoped that research in these areas could result in “easier and fairer [recruitment processes], because it does always feel like people with Asperger's and autism are at a disadvantage” (P079).

#### Theme 2: organizational culture

##### Exploring stigma, discrimination, and exploitation in the workplace

As a result of differences between them and their non-autistic colleagues, many autistic people reported experiencing stigma, discrimination, and exploitation in the workplace. For example, one participant explained:

Employers have the habitual predisposition to make use of hyperfocus, it produces excellent results … Unfortunately, in my experience, this is an opportunity for exploitation. Management [and] colleagues will often take advantage of my work ethic and weak social skills, i.e., not understanding when to say no. This has occurred in every job I've had. (P167)

Participants wanted research to quantify the extent to which autistic people face such experiences of stigma, discrimination, and exploitation (“[research] the level of stigma and discrimination autistic people encounter in the workplace,” P037) and to determine “how far discrimination is responsible for the appallingly low rates of employment among autistic people” (P008). Relatedly, participants expressed a need for research to contribute to the development of “interventions and schemes (and not just compulsory e-learning) which reduce the likelihood of bullying of autistic people in the workplace” (P113). Ultimately, participants hoped that research could help to “remove the judgement and discrimination” (P181) that autistic people face in the workplace, and “drive changes that make autistic employees feel welcomed, valued and advocated for” (P171).

##### Improving understanding and acceptance from others

Participants repeatedly reported finding the predominantly neurotypical norms and expectations of the workplace confusing and difficult to navigate. Participants highlighted differences in communication as a pertinent barrier to successful employment: “I was diagnosed with Asperger's due to a long-term pattern in losing my job. It was never due to my work being bad … but [because] I'd offended someone/said the wrong thing/looked at someone the wrong way” (P042).

As such, participants called for research to “question the neurotypical style and methods of communication as being the ‘default’ against which autistic people are judged” (P077) and “raise better awareness [of autism]” (P191). They felt such research could result in a “better understanding amongst neurotypicals of how to make autistic employees feel included and how to interact with us effectively” (P129).

Practically, some participants hoped autism-employment research could contribute to the development of “up-to-date autism training which challenges stereotypes” (P013), including, for example, by providing employers with information regarding the strengths of autistic employees (“it would be excellent if neurotypical employers became more aware of what autistic people have to offer and accept them as being of equal worth,” P018) and how these strengths could be harnessed: “it would be great if researchers would be able to prove that employing people on the spectrum is beneficial and how” (P022).

Participants felt, however, that it was important for research to evaluate the impact of such training (“does autism training improve autistic people's experiences in the workplace?,” P152) to ensure such education leads to meaningful change: “training can be given to give people knowledge but how do we measure if their attitudes have changed?” (P156). Some felt that research should also result in more practical support to help autistic people navigate the predominantly neurotypical workplace: “[I would like to see] training programs for autistic students about office politics and networking skills, and how they can use these to advance their careers” (P163).

##### Better networks of support

A common method autistic adults used to alleviate work-related stress, and thus sustain employment, was support from colleagues, advocates, and champions: “things like workplace coaching and mentoring help to keep people in jobs” (P143). They felt it was important for research to show autistic people “they have available resources and services to protect them in the workplace” (P053). Nevertheless, many felt that existing support was lacking and indicated this may be a fruitful area for research to explore: “[I would like to see research on] how to best support professionals in the workplace. I can find quite a bit of information about starting jobs or support in non-professional level jobs but not that much that's been helpful for me” (P123).

##### Access to workplace adjustments

Many participants perceived workplace adjustments as an integral component in supporting autistic people to sustain employment. Yet, many participants reported being “made to feel inadequate” (P029) for requesting adjustments or felt that organizations were simply offering adjustments “to earn some sort of invisible ‘good company’ points” (P088). Indeed, one participant noted that “wheelchair users need ramps, wide doors, disabled bays etc. and this is now implemented as law” (P010) but felt that equal measures were not in place for people with more “hidden” disabilities.

As such, participants said it may be beneficial for research to identify the workplace adjustments that may be particularly beneficial for autistic people: “[I would like research to identify] what accommodations are the most widely used [and] most effective” (P039), so autistic people can advocate for the adjustments they may require. Yet, others felt it was important for employers to take ownership of implementing adjustments: “It shouldn't be about putting all the onus on autistic people. We are already made to feel that everything we do is putting people out” (P038). As such, participants wanted research to demonstrate the wide-reaching benefits of adjustments to encourage employers to improve implementation:

Quantifying how [productivity] improves when adjustments are made would also be very helpful. Being able to tell a company that they can expect a certain percentage improvement in productivity by making adjustments is very powerful (P188).

#### Theme 3: the employment journey

##### Experiences of career progression

While participants appreciated the prospect of employment research that aligns with their priorities, many indicated that current autism-employment research lacks “the whole life perspective” (P195). This gap left participants with many unanswered questions about autism and employment, for example: “what do autistic careers/professional lives/jobs over a lifetime look like?” (P195) and “what does successful work look like for autistic adults?” (P191).

Unfortunately, many participants were concerned that autistic people may not be given the same chances to progress within their careers as non-autistic people: “so many of us are underemployed because we can't do workplace politics or read people well enough to get along” (P128). They expressed explicit concerns about “the impact of ‘being different’ on [the likelihood of receiving a] promotion” (P087). Accordingly, participants wanted research to address questions such as “do people on the spectrum get paid fairly? [And] do we get promoted at the same rate?” (P034).

Indeed, some participants highlighted that many senior positions entail management responsibilities, and perceived such responsibilities to be “very difficult, time consuming and tiring” (P023). As such, participants wanted research to result in “more guidance about how autistic people can successfully progress” (P127). Conversely, some participants felt that there are likely many “undiagnosed autistic people [who] have successful careers” (P008) and wanted research to highlight more successful case studies to “figure out what went right. How did they find a rewarding career that didn't involve playing the corporate game of thrones? What career guidance did they get? Where did they find out about the opportunities they took?” (P103).

##### Transitions out of employment

Participants also indicated that transitions out of employment may be particularly challenging, although common, for autistic people. Relatedly, participants reported wanting more research aiming to understand such transitions, including “how did the job end? Why did the job end?” (P102) and “the experiences of autistic people who have lost their job as a result of their autism” (P048). Participants also highlighted a need for more research examining the transition to retirement, and how autistic people can be best supported in “preparing for retirement” (P195).

#### Theme 4: the negative impact of non-inclusive workplace environments

##### Mental-health related consequences of employment

While employment was a desirable outcome for many participants, employment was also felt to negatively impact people “physically, mentally, and emotionally” (P062), with participants reporting specific “burnout issues related to work” (P074). Accordingly, participants reported a need for research to highlight “the effect of corporate culture on mental health” (P021) and “inform changes to enable all autistic people to be able to work if they wish … in physical and cultural work environments that have a positive—rather than detrimental—effect upon their mental health” (P116).

##### Workplace masking as a perceived necessity

Participants also discussed the perceived need to mask in the workplace and expressed concerns about “the toll it takes” (P063). They reported that they “would like to feel that it is not so necessary to have to mask in the workplace” (P021) and called for research to “make it so we don't have to mask, so we can have a job and feel like we are being part of the world like everyone else” (P019). One participant noted, however, that “despite the stresses of masking, I think it is important to try to fit in, and maybe more people could secure and maintain employment if they did. Perhaps this needs to be looked at more?” (P095).

#### Theme 5: wider reflections on autism-employment research

##### The need for coproduced autism-employment research

Participants made important reflections about the approach researchers should take to autism-employment research, and the importance of involving the autistic community at the heart of research. For example, many highlighted the importance of “utilising the experiences of [those with lived experience] as key employed researchers” (P002) and explained that “involvement of autistic people at every stage of the research is critical. We are the experts on autism, and too often our voices are ignored” (P077). Participants noted that involvement in research should extend beyond simple participation and should involve autistic people “as co-researchers or advisors … ask autistic people about their research priorities, like you are doing” (P175).

##### The importance of including seldom-heard groups within autism-employment research

Participants also indicated the importance of exploring the workplace experiences of autistic people that are often missed in autism research. For example, one participant reflected how “undiagnosed autistic adults … those who have learned to mask … [and] those who are not in mental health crisis are underrepresented” (P147). Accordingly, participants felt “a bit more balance” (P023) in future research would be an important step to ensure findings are relevant for as many autistic people as possible.

## Discussion

In this study, we sought to identify a set of specific and actionable priority areas for employment-related autism research, according to autistic people, and to understand *why* these topics were prioritized. Our findings were in line with the findings of a recent consultation in Australia,^19^ highlighting a broader need for research to identify ways in which we can support autistic people to gain and sustain suitable employment (e.g., through workplace adjustments and inclusive organizational culture). Unique to this study was the identification of a need for more research exploring later stages of the employment journey, such as experiences of career progression and transitioning out of employment. Importantly, participants in this study also highlighted *how* we should conduct such research: working directly with autistic people as co-researchers and ensuring the whole autistic population is well represented. Later, we discuss the specific topic areas that our participants wanted research to cover, with reference to existing literature and potential avenues for future research.

The first topic identified by our participants was autistic people's access to employment. Specifically, participants wanted research to (a) highlight current opportunities for suitable employment, such as autism-specific employment opportunities;^48–50^ (b) explore autistic people's experiences of disclosing their diagnosis^31–33^ (see Lindsay et al.^34^ for a review); (c) explore the impact of having an intersectional identity (e.g., minority gender/ethnicity/socioeconomic background, *and* being autistic) on employment experiences (e.g., see Eilenberg et al.^51^ and Hayward et al.^52^ for relevant systematic reviews); and (d) highlight how to improve hiring processes.^53–55^ Encouragingly, research aiming to improve autistic people's access to employment appears to be growing, with a wide range of initiatives, driven by researcher-industry partnerships, aiming to support autistic people in obtaining employment.^56–60^ Yet, there are limited high-quality evaluations of such initiatives. Indeed, a recent systematic review examining the outcomes of employment programs for autistic people highlighted several methodological limitations with existing studies and concluded, “the low number [of studies] included in the final sample indicates that there is a specific need for high-quality studies using rigorous methodologies”^61(p. 13)^As such, future research should seek to conduct more rigorous testing of such initiatives to establish which, if any, achieve the outcomes they purport to achieve, and thus identify the most effective ways of supporting autistic people to obtain employment.

The second topic identified by participants focused on the need for research to consider not just employment rates but also the *sustainability* of employment as it relates directly to autistic people. Our participants highlighted that organizational culture often acts as a barrier to sustainable employment for autistic people. Participants, therefore, wanted more research that might lead to meaningful organizational change. Examples of suggested research topics included: (a) exploring autistic employees' experiences of stigma and discrimination at work;^37–39^ (b) developing and evaluating interventions to improve non-autistic employees' attitudes toward autistic people;^62^ (c) examining and evaluating how autistic people can be better supported at work (e.g., through supported employment, mentors, and/or job-coaches);^21,63,64^ and (d) identifying which workplace adjustments may be useful for autistic people^35^ (see Khalifa et al.^36^ and Lindsay et al.^34^ for previous reviews). While research in this area is progressing, once again, the evidence base remains somewhat underdeveloped. In particular, there appears to be a paucity of research that goes beyond describing people's employment experiences and moves toward the development and, importantly, evaluation of programs that make a meaningful difference to autistic people's employment experiences—especially at the level of organizational culture.

One proposed method for facilitating such change is the use of autism-specific training programs.^34,65,66^ Currently, however, there remain a few empirical evaluations of the effectiveness of workplace disability-training programs.^67,68^ Indeed, a recent systematic review examining human resource strategies (e.g., training initiatives) for improving the inclusion of people with disabilities in employment stated that “many questions remain open including the most effective training design and content… future research should conduct formal training evaluations, using longitudinal and randomized field studies.”^68(p. 75)^ To our knowledge, only one evaluation of an autism-specific training intervention has been conducted.^62^ The development and empirical testing of autism-specific workplace training programs to improve non-autistic attitudes toward autistic people should, therefore, be a key avenue for future research.

Third, our participants said they wanted more research to examine the full employment lifecycle, not just research on the early stages of one's career. They highlighted autistic people's experiences of career progression and transitioning out of employment (e.g., due to termination of employment, or retirement) as key topics for future research. They also identified aspects of employment that may be particularly challenging for autistic people, including potentially unique barriers related to career progression, such as poor job matching by job coaches and/or employers.^69,70^ Indeed, research suggests that the underemployment (i.e., holding a job below one's skill level or otherwise capacity) of autistic people is vast, with underemployment rates reportedly between ∼20% and 46%.^40–42^ Yet, seemingly minimal attention has been paid to addressing this disparity. To our knowledge, no research has directly examined autistic people's experiences of career progression, and only one paper has explored, using a single case study design, an autistic person's experience of the transition from work to retirement.^71^ The dearth of research in this area makes it a worthy candidate for future research.

Finally, participants discussed at length the negative impact that non-inclusive workplace environments have on autistic employees and said they wanted more research in this regard. Indeed, many participants reported facing stigma, discrimination, and underemployment, and perceived such experiences as a direct result of being autistic. Consequently, many participants felt they had to mask their authentic selves to succeed at work. Such negative employment experiences were felt to have serious ramifications for mental health and well-being.

While masking in the workplace may be, to some extent, common across all neurotypes, emerging evidence suggests that autistic people face unique pressures to mask, and workplace masking has been found to have more severe consequences for mental health than masking in other contexts (Pryke-Hobbes et al., “The workplace masking experiences of autistic, non-autistic neurodivergent, and neurotypical adults in the UK” unpublished manuscript). In the general population, the benefits of employment for mental health, well-being, and quality of life are well established.^72–76^ However, there is limited, weak, and often conflicting, evidence of an association between (un)employment and mental health and/or quality of life in the autistic population.^77–80^ Based on our findings, we suggest that the lack of a clear association in this regard may be due to the disproportionate number of autistic people facing negative workplace experiences such as stigma, discrimination, and the resulting need to mask their true selves. Indeed, while a recent study^22^ examined the association between job satisfaction and mental health and well-being, no research, to our knowledge, has examined the potential impact of *poor* workplace experiences on autistic people's mental health. This may be an important avenue for future research, especially as autistic people are more likely to experience co-occurring mental health conditions than the general population.^81^

In addition to identifying key autism-employment research priorities, our participants provided invaluable insights regarding *how* researchers should seek to conduct such research. One key issue in this regard was the importance of listening to, and including, seldom-heard groups within autism research. Indeed, some of our participants suggested it may not be uncommon for research to involve the same autistic people, or for research samples to fail to represent the diversity that exists within the autistic population. These claims are not unfounded: evidence suggests that autistic people with an intellectual disability, and those from ethnic minority backgrounds are all-too-often overlooked in current autism research.^82–84^ Similarly, and perhaps somewhat inevitably, research may fail to reach those who are not interested in the research topic, or participating in research in general, as well as autistic people for whom being autistic is not central to their identity. As such, our participants wanted researchers to make concerted efforts for more diverse participant samples, so that they could make more meaningful recommendations for different groups of autistic people.

The priority topics identified in this study map onto the foci of much recent autism-employment research. While this is encouraging, it also suggests that the findings from existing autism-employment research are not necessarily being felt “on the ground”—due to ineffectual dissemination strategies and/or a blockage in the “translational pipeline.”^12^ The former is perhaps unsurprising given that research findings are typically published in academic journals, making them inaccessible (or behind a paywall) to most of the population.^85^ Access alone (e.g., making articles open-access), however, may not be enough to aide dissemination of research findings, as research is “often linguistically inaccessible” (p. 1089) to the general public.^86^ Autism-employment research is unlikely to have the intended impact on employment practices if the findings are ineffectively disseminated. Adopting a participatory approach to employment research should ensure that research is disseminated in a more accessible and meaningful way, thus making it more likely to be translated in practice.

Based on our findings, we make three key recommendations. First, we suggest that researchers should involve autistic people in each aspect of the autism-employment research process, from conceptualization to data collection, analysis, and dissemination. While the involvement of autistic people in autism research is gradually becoming more commonplace, genuine co-production whereby autistic people and non-autistic researchers share power in the research decision-making process is still rare, or rarely reported.^11,12,87–89^ Indeed, evidence suggests that autism researchers may lack a genuine understanding of participatory research^87^ and that the nature of academic research (e.g., funding constraints) could preclude the meaningful involvement of autistic people.^90^ Overcoming these barriers could involve tailored training for autism researchers at any stage of their career, as well as more systemic changes to research processes to allow for the increased time and cost of participatory research.^90^

Second, we suggest that autism researchers make greater efforts to reach people who are currently under-served in autism research. Such efforts would mean actively engaging with marginalized groups within the autistic and autism communities, including those with minority traits and/or characteristics. Researchers should also be transparent in the reporting of participant characteristics and acknowledge the limitations of their sample.

Finally, autism-employment research would benefit from improved science communication efforts to ensure research findings reach the community, and result in meaningful and tangible change.^12^ At the micro level, this may involve working with community partners to develop new, accessible ways to disseminate research findings such as research toolkits, accessible videos, and/or podcasts (see Crane et al.,^91^ Heasman et al.^92^ and Whitehouse^93^). At the macro level, this could involve systemic change to make research easier to access; for example, by allowing equal access to research (e.g., through open science practices), and by making accessible science communication mandatory (e.g., using lay language).

### Limitations

Most notably, our own sample was not representative of the autistic population. For example, most of our participants were employed and educated to a Bachelor's Degree level or higher. Similarly, many more autistic women chose to take part than autistic men. While this pattern of gender distribution is not uncommon in survey research^94^, it does go against current population estimates, which suggest a 3:1 male to female ratio for autism diagnoses.^95^

While the sample was fairly representative in terms of ethnicity (77.7% were White, compared with 81.7% in the 2021 Census^96^), people from minority ethnic backgrounds are likely to have unique experiences of employment,^97^ and may therefore have alternative priorities for future research. Similarly, given that our participants were required to complete a survey reflecting on their experiences, and considering future avenues for research, autistic people with intellectual disabilities were likely precluded from participating in this research. Yet, those with an intellectual disability are also likely to have unique experiences of employment^98^ and may have alternative research priorities.

As a result, the research priorities outlined earlier only reflect the priorities of a sub-group of the autistic population and not those of all autistic people. It is also worth noting that, unlike other priority-setting exercises,^19^ we did not ask participants to rank their priorities, which means we are unable to ascertain which of the identified priorities are the most pressing for autistic people.

## Conclusions

Despite the outlined limitations, this study clearly outlined the priorities of autistic people for autism-employment research moving forward. We hope that, by clearly presenting what the autistic community needs and wants from employment research, researchers will conduct more studies in response to and in close collaboration with autistic people, who should have the power to drive research that aims to benefit them.

## Supplementary Material

Supplemental data

## Supplementary Material

Supplementary Appendix Table SA1

## Notes

^*^
Participants were only able to select one response option to the question regarding employment status. Those that fit into multiple categories (e.g., student and employed) were asked to select the option they believed most accurately represented their employment status.

## References

[1] Pellicano E, Dinsmore A, Charman T. A Future Made Together: Shaping Autism Research in the UK. London: Institute of Education; 2013.

[2] Warner G, Cooper H, Cusack J. A review of the autism research funding landscape in the United Kingdom. Autistica. https://www.autistica.org.uk/downloads/files/Autistica-Scoping-Report.pdf Accessed December 16, 2022.

[3] Interagency Autism Coordinating Committee. IACC/OARC autism spectrum disorder publications analysis: The global landscape of autism research. Interagency Autism Coordinating Committee, US Department of Health and Human Services. https://iacc.hhs.gov/publications/publications-analysis/2012/publications_analysis_2012.pdf Accessed December 16, 2022.

[4] Daniels SA, Warner G. An International Autism Portfolio Analysis by United States, United Kingdom, and Canada. International Society for Autism Research. Rotterdam, Netherlands: Office of Autism Research Coordination; May 2018.

[5] den Houting J, Pellicano E. A portfolio analysis of autism research funding in Australia, 2008–2017. J Autism Dev Disord. 2019;49(11):4400–4408. 10.1007/s10803-019-04155-1 31375971

[6] Harris L, Gilmore D, Longo A, Hand BN. Patterns of US federal autism research funding during 2017–2019. Autism. 2021;25(7):2135–2139. 10.1177/13623613211003430 33765838

[7] Office of Autism Research Coordination, National Institute of Mental Health, on behalf of the Interagency Autism Coordinating Committee. 2016 International Autism Spectrum Disorder Research: Portfolio Analysis Report. https://iacc.hhs.gov/publications/international-portfolio-analysis/2016/portfolio_analysis_2016.pdf Accessed December 16, 2022.

[8] Autistica. Your Questions: Shaping Future Autism Research. https://www.autistica.org.uk/downloads/files/Autism-Top-10-Your-Priorities-for-Autism-Research.pdf Accessed December 16, 2022.

[9] Frazier TW, Dawson G, Murray D, Shih A, Sachs JS, Geiger A. Brief report: A survey of autism research priorities across a diverse community of stakeholders. J Autism Dev Disord. 2018;48(11):3965–3971. 10.1007/s10803-018-3642-6 29948533

[10] Gatfield O, Mangan C, Harr T, Kinniburgh A, Rodger S. 2016 Autism research priorities survey report. Autism CRC. https://www.autismcrc.com.au/sites/default/files/inline-files/Research%20priorities%20survey%20-%20Final%20report.pdf Accessed December 16, 2022.

[11] Pellicano E, Dinsmore A, Charman T. What should autism research focus upon? Community views and priorities from the United Kingdom. Autism. 2014;18(7):756–770. https://doir.org/10.1177/1362361314529627 24789871 10.1177/1362361314529627 PMC4230972

[12] Pellicano E, Dinsmore A, Charman T. Views on researcher-community engagement in autism research in the United Kingdom: A mixed-methods study. PLoS One. 2014;9(10):e109946. https://doi.org10.1371/journal.pone.0109946 25303222 10.1371/journal.pone.0109946 PMC4193852

[13] Clark M, Adams D. Listening to parents to understand their priorities for autism research. PloS One. 2020;15(8):e0237376. 10.1371/journal.pone.0237376 32790720 PMC7425861

[14] Rudan I. Setting health research priorities using the CHNRI method: IV. Key conceptual advances. *J Glob Health*. 2016;6(1):010501. 10.7189/jogh-06-010501 PMC4938380 27418959

[15] Tomlinson M, Yasamy MT, Emerson E, Officer A, Richler D, Saxena S. Setting global research priorities for developmental disabilities, including intellectual disabilities and autism. J Intellect Disabil Res. 2014;58(12):1121–1130. 10.1111/jir.12106 24397279 PMC4556421

[16] Roche L, Adams D, Clark M. Research priorities of the autism community: A systematic review of key stakeholder perspectives. Autism. 2021;25(2):336–348. 10.1177/1362361320967790 33143455

[17] Gotham K, Marvin AR, Taylor JL, et al. Characterizing the daily life, needs, and priorities of adults with autism spectrum disorder from Interactive Autism Network data. Autism. 2015;19(7):794–804. 10.1177/1362361315583818 25964655 PMC4581903

[18] Australian Autism Research Council. Draft Research Priorities for Consultation 2019. https://www.autismcrc.com.au/sites/default/files/inline-files/AARC_Draft-Research-Priorities-for-Consultation-2019.pdf Accessed December 16, 2022.

[19] Australian Autism Research Council. Research report on focus groups to identify research questions for community informed priority areas. https://www.autismcrc.com.au/sites/default/files/aarc/AARC_Research_Report_Focus_Groups_Final_Report.pdf Accessed December 16, 2022.

[20] Poulsen R, Brownlow C, Lawson W, Pellicano E. Meaningful research for autistic people? Ask autistics!. Autism. 2022;26(1):3–5. 10.1177/13623613211064421 35000419

[21] Nicholas DB, Zwaigenbaum L, Zwicker J, et al. Evaluation of employment-support services for adults with autism spectrum disorder. Autism. 2018;22(6):693–702. 10.1177/1362361317702507 28637355

[22] Hedley D, Uljarević M, Bury SM, Dissanayake C. Predictors of mental health and well-being in employed adults with autism spectrum disorder at 12-month follow-up. Autism Res. 2019;12(3):482–494. 10.1002/aur.2064 30675764

[23] Umagami K, Remington A, Lloyd-Evans B, Davies J, Crane L. Loneliness in autistic adults: A systematic review. Autism. 2022;26(8):2117–2135. 10.1177/13623613221077721 35257592 PMC9597154

[24] Office for National Statistics. Outcomes for disabled people in the UK: 2020. Office for National Statistics; UK Statistics Agency. https://www.ons.gov.uk/peoplepopulationandcommunity/healthandsocialcare/disability/articles/outcomesfordisabledpeopleintheuk/2020#employment Accessed December 16, 2022.

[25] Zwicker J, Zaresani A, Emery JH. Describing heterogeneity of unmet needs among adults with a developmental disability: An examination of the 2012 Canadian Survey on Disability. Res Dev Disabil. 2017;65:1–11. 10.1016/j.ridd.2017.04.003 28412577

[26] Australia Bureau of Statistics. Disability, Ageing and Carers, Australia: Summary of Findings, 2015. Australia Bureau of Statistics. https://www.abs.gov.au/ausstats/abs@.nsf/Latestproducts/4430.0Main%20Features752015 Accessed December 16, 2022.

[27] Gerhardt PF, Lainer I. Addressing the needs of adolescents and adults with autism: A crisis on the horizon. J Contemp Psychother. 2011;41(1):37–45. 10.1007/s10879-010-9160-2

[28] López B, Keenan L. Barriers to employment in autism: Future challenges to implementing the Adult Autism Strategy. Autism Research Network 2014;1–17.

[29] Nicholas DB, Hedley D, Randolph J K, Raymaker DM, Robertson SM, Vincent J. An expert discussion on employment in autism. Autism Adulthood. 2019;1(3):162–169. 10.1089/aut.2019.29003.djn 35252769 PMC8890031

[30] Roux AM, Shattuck PT, Cooper BP, Anderson KA, Wagner M, Narendorf SC. Postsecondary employment experiences among young adults with an autism spectrum disorder. J Am Acad Child Adolesc Psychiatry. 2013;52(9):931–939. 10.1016/j.jaac.2013.05.019 23972695 PMC3753691

[31] Huang Y, Hwang YIJ, Arnold SR, Lawson LP, Richdale AL, Trollor JN. Autistic adults' experiences of diagnosis disclosure. J Autism Dev Disord. 2022;52(12):5301–5307. 10.1007/s10803-021-05384-z 34978025

[32] Romualdez AM, Heasman B, Walker Z, Davies J, Remington A. “People might understand me better”: Diagnostic disclosure experiences of autistic individuals in the workplace. Autism Adulthood. 2021;3(2):157–167. 10.1089/aut.2020.0063

[33] Romualdez AM, Walker Z, Remington A. Autistic adults' experiences of diagnostic disclosure in the workplace: Decision-making and factors associated with outcomes. Autism Dev Lang Impair. 2021;6:1–12. 10.1177/23969415211022955 PMC9620671 36381532

[34] Lindsay S, Osten V, Rezai M, Bui S. Disclosure and workplace accommodations for people with autism: A systematic review. Disabil Rehabil. 2021;43(5):597–610. 10.1080/09638288.2019.1635658 31282214

[35] Davies J, Heasman B, Livesey A, Walker A, Pellicano E, Remington A. Autistic adults' views and experiences of requesting and receiving workplace adjustments in the UK. PLoS One. 2022;17(8):e0272420. 10.1371/journal.pone.0272420 35930548 PMC9355205

[36] Khalifa G, Sharif Z, Sultan M, Di Rezze B. Workplace accommodations for adults with autism spectrum disorder: A scoping review. Disabil Rehabil. 2020;42(9):1316–1331. 10.1080/09638288.2018.1527952 30714420

[37] Doyle N, McDowall A, Waseem U. Intersectional stigma for Autistic people at work: A compound adverse impact effect on labor force participation and experiences of belonging. Autism Adulthood. 2022;4(4):340–356. 10.1089/aut.2021.0082 36777372 PMC9908290

[38] Johnson TD, Joshi A. Dark clouds or silver linings? A stigma threat perspective on the implications of an autism diagnosis for workplace well-being. J Appl Psychol. 2016;101(3):430–449. 10.1037/apl0000058 26595753

[39] Lorenz T, Brüning CR, Waltz M, Fabri M. Not a stranger to the dark: Discrimination against autistic students and employees. Adv Autism. 2021;7(1):60–72. 10.1108/AIA-10-2019-0036

[40] Baldwin S, Costley D, Warren A. Employment activities and experiences of adults with high-functioning autism and Asperger's disorder. J Autism Dev Disord 2014;44(10):2440–2449. 10.1007/s10803-014-2112-z 24715257

[41] Harvery M, Froude EH, Foley KR, Trollor JN, Arnold SR. Employment profiles of autistic adults in Australia. Autism Res. 2021;14(10):2061–2077. 10.1002/aur.2588 34374491

[42] Ohl A, Grice Sheff M, Small S, Nguyen, J, Paskor K, Zanjirian A. Predictors of employment status among adults with Autism Spectrum Disorder. Work. 2017;56(2):345–355. 10.3233/WOR-172492 28211841

[43] Nicholas DB, Hodgetts S, Zwaigenbaum L, et al. Research needs and priorities for transition and employment in autism: Considerations reflected in a “Special Interest Group” at the International Meeting for Autism Research. Autism Res. 2017;10(1):15–24. 10.1002/aur.1683 27753278

[44] Shattuck PT, Lau L, Anderson KA, Kuo AA. A national research agenda for the transition of youth with autism. Pediatrics. 2018;141(Suppl 4):S355–S361. 10.1542/peds.2016-4300M 29610417

[45] Braun V, Clarke V. Successful Qualitative Research: A Practical Guide for Beginners. London, UK: Sage; 2013.

[46] Braun V, Clarke V. Reflecting on reflexive thematic analysis. Qual Res Sport Exerc Health. 2019;11(4):589–597. 10.1080/2159676X.2019.1628806

[47] Byrne D. A worked example of Braun and Clarke's approach to reflexive thematic analysis. Qual Quant. 2022;56(3):1391–1412. 10.1007/s11135-021-01182-y

[48] Grenawalt TA, Brinck EA, Kesselmayer RF, et al. Autism in the workforce: A case study. J Manage Organ. 2020:1–16. 10.1017/jmo.2020.15

[49] Kesselmayer RF, Ochrach CM, Phillips BN, et al. Autism employment initiative in a global business management consultancy firm: A case study. Rehabil Couns Educ J. 2022;11(1):1–11. 10.52017/001c.32416

[50] Remington A, Pellicano E. ‘Sometimes you just need someone to take a chance on you’: An internship programme for autistic graduates at Deutsche Bank, UK. J Manage Organ. 2019;25(4):516–534. 10.1017/jmo.2018.66

[51] Eilenberg JS, Paff M, Harrison AJ, Long KA. Disparities based on race, ethnicity, and socioeconomic status over the transition to adulthood among adolescents and young adults on the autism spectrum: A systematic review. Curr Psychiatry Rep. 2019;21(5):32. 10.1007/s11920-019-1016-1 30903399

[52] Hayward SM, McVilly KR, Stokes MA. Challenges for females with high functioning autism in the workplace: A systematic review. J Disabil Rehabil. 2018;40(3):249–258. 10.1080/09638288.2016.1254284 27927031

[53] Davies J, Heasman B, Livesey A, Walker A, Pellicano E, Remington A. Access to employment: A comparison of autistic and non-autistic adults' recruitment experiences in the UK. Autism. 2023:1–18. 10.1177/13623613221145377 PMC10375005 36597955

[54] Maras K, Norris JE, Nicholson J, Heasman B, Remington A, Crane L. Ameliorating the disadvantage for autistic job seekers: An initial evaluation of adapted employment interview questions. Autism. 2021;25(4):1060–1075. 10.1177/1362361320981319 33339462 PMC8108109

[55] Vincent J. Employability for UK university students and graduates on the autism spectrum: Mobilities and materialities. Scand J Disabil Res. 2020;*22*(1):12–24. 10.16993/sjdr.656

[56] Munandar VD, Bross LA, Zimmerman KN, Morningstar ME. Video-based intervention to improve storytelling ability in job interviews for college students with autism. Career Dev Transit Except Individ. 2021;44(4):203–215. 10.1177/2165143420961853

[57] Strickland DC, Coles CD, Southern LB. JobTIPS: A transition to employment program for individuals with autism spectrum disorders. J Autism Dev Disord. 2013;43(10):2472–2483. 10.1007/s10803-013-1800-4 23494559 PMC3706489

[58] Wehman PH, Schall CM, McDonough J, et al. Competitive employment for youth with autism spectrum disorders: Early results from a randomized clinical trial. J Autism Dev Disord 2014;44(3):487–500. 10.1007/s10803-013-1892-x 23893098

[59] Wehman P, Schall C, McDonough J, et al. Project SEARCH for youth with autism spectrum disorders: Increasing competitive employment on transition from high school. J Posit Behav Interv. 2013;15(3):144–155. 10.1177/1098300712459760

[60] Wehman P, Schall C, McDonough J, et al. Competitive employment for transition-aged youth with significant impact from autism: A multi-site randomized clinical trial. J Autism Dev Disord. 2020;50(6):1882–1897. 10.1007/s10803-019-03940-2 30825082

[61] Fong CJ, Taylor J, Berdyyeva A, et al. Interventions for improving employment outcomes for persons with autism spectrum disorders: A systematic review update. Campbell Syst Rev. 2021;17(3):e1185. 10.1002/cl2.1185 37052419 PMC8354554

[62] Scott M, Falkmer M, Falkmer T, Girdler S. Evaluating the effectiveness of an autism-specific workplace tool for employers: A randomised controlled trial. J Autism Dev Disord. 2018;48(10):3377–3392. 10.1007/s10803-018-3611-0 29767376

[63] Müller E, Schuler A, Burton B, Yates GB. Meeting the vocational support needs of individuals with Asperger syndrome and other autism spectrum disabilities. J Vocat Rehabil. 2003;18:163–175.

[64] Vogeley K, Kirchner JC, Gawronski A, et al. Toward the development of a supported employment program for individuals with high-functioning autism in Germany. Eur Arch Psychiatry Clin Neurosci. 2013;263(Suppl 2):S197–S203. 10.1007/s00406-013-0455-7 24077909

[65] Hendricks D. Employment and adults with autism spectrum disorders: Challenges and strategies for success. J Vocat Rehabil. 2010;32(2):125–134. 10.3233/JVR-2010-0502

[66] Petty S, Tunstall L, Richardson H, Eccles N. Workplace adjustments for autistic employees: What is ‘reasonable’? J Autism Dev Disord. 2023;53(1):236–244. 10.1007/s10803-021-05413-x 35020116 PMC8752384

[67] Phillips BN, Deiches J, Morrison B, et al. Disability diversity training in the workplace: Systematic review and future directions. J Occup Rehabil. 2016;26(3):264–275. 10.1007/s10926-015-9612-3 26519035

[68] Schloemer-Jarvis A, Bader B, Böhm SA. The role of human resource practices for including persons with disabilities in the workforce: A systematic literature review. Int J Hum Resour Manage. 2022;33(1):45–98. 10.1080/09585192.2021.1996433

[69] Raymaker DM, Sharer M, Maslak J, et al. “[I] don't wanna just be like a cog in the machine”: Narratives of autism and skilled employment. Autism. 2023;27(1):65–75. 10.1177/13623613221080813 35362339 PMC9525447

[70] Sharpe M, Hutchinson C, Alexander J. The lived experiences and perspectives of people with autism spectrum disorder in mainstream employment in Australia. Disabilities. 2022;2(2):164–177. 10.3390/disabilities2020013

[71] Hodges JS, Luken K, Hubbard A. Supporting the transition of one man with autism from work to retirement. Ther Recreation J. 2004;38(3):301–311.

[72] Amiri S. Unemployment associated with major depression disorder and depressive symptoms: A systematic review and meta-analysis. Int J Occup Saf Ergon. 2022;28(4):2080–2092. 10.1080/10803548.2021.1954793 34259616

[73] Backhans MC, Hemmingsson T. Unemployment and mental health—Who is (not) affected? Eur J Public Health. 2012;22(3):429–433. 10.1093/eurpub/ckr059 21602222

[74] Heinz AJ, Meffert BN, Halvorson MA, et al. Employment characteristics, work environment, and the course of depression over 23 years: Does employment help foster resilience? Depress Anxiety. 2018;35(9):861–867. 10.1002/da.22782 29878482 PMC6123281

[75] Voßemer J, Gebel M, Täht K, et al. The effects of unemployment and insecure jobs on well-being and health: The moderating role of labor market policies. Soc Indic Res. 2018;138(3):1229–1257. 10.1007/s11205-017-1697-y

[76] Wulfgramm M. Life satisfaction effects of unemployment in Europe: The moderating influence of labour market policy. J Eur Soc Policy. 2014;24(3):258–272. 10.1177/0958928714525817

[77] Barneveld PS, Swaab, H, Fagel, S, et al. Quality of life: A case-controlled long-term follow-up study, comparing young high-functioning adults with autism spectrum disorders with adults with other psychiatric disorders diagnosed in childhood. Compr Psychiatry. 2014;55(2):302–310. 10.1016/j.comppsych.2013.08.001 24290884

[78] Billstedt E, Gillberg IC, Gillberg C. Aspects of quality of life in adults diagnosed with autism in childhood: A population-based study. Autism. 2011;15(1):7–20. 10.1177/1362361309346066 20923888

[79] García-Villamisar D, Wehman P, Navarro MD. Changes in the quality of autistic people's life that work in supported and sheltered employment. A 5-year follow-up study. *J Vocat Rehabil*. 2002;17(4):309–312.

[80] Lin LY, Huang PC. Quality of life and its related factors for adults with autism spectrum disorder. Disabil Rehabil. 2019;41(8):896–903. 10.1080/09638288.2017.1414887 29228834

[81] Lai M C, Kassee C, Besney R, et al. Prevalence of co-occurring mental health diagnoses in the autism population: A systematic review and meta-analysis. Lancet Psychiatry. 2019;6(10):819–829. 10.1016/S2215-0366(19)30289-5 31447415

[82] Hilton CL, Fitzgerald RT, Jackson KM, et al. Brief report: Under-representation of African Americans in autism genetic research: A rationale for inclusion of subjects representing diverse family structures. J Autism Dev Disord. 2010;40(5):633–639. 10.1007/s10803-009-0905-2 19936905 PMC3645854

[83] Russell G, Mandy W, Elliott D, White R, Pittwood T, Ford T. Selection bias on intellectual ability in autism research: A cross-sectional review and meta-analysis. Mol Autism. 2019;10(1):9. 10.1186/s13229-019-0260-x 30867896 PMC6397505

[84] West EA, Travers JC, Kemper TD, et al. Racial and ethnic diversity of participants in research supporting evidence-based practices for learners with autism spectrum disorder. J Spec Educ. 2016;50(3):151–163. 10.1177/0022466916632495

[85] Piwowar H, Priem J, Larivière V, et al. The state of OA: A large-scale analysis of the prevalence and impact of Open Access articles. *PeerJ*, 2018;6:e4375. 10.7717/peerj.4375 29456894 PMC5815332

[86] Bertilsdotter Rosqvist H, Kourti M, Jackson-Perry D, et al. Doing it differently: Emancipatory autism studies within a neurodiverse academic space. Disabil Soc. 2019;34(7–8):1082–1101. 10.1080/09687599.2019.1603102

[87] den Houting J, Higgins J, Isaacs K, Mahony J, Pellicano E. ‘I'm not just a guinea pig’: Academic and community perceptions of participatory autism research. Autism. 2021;25:148–163. 10.1177/1362361320951696 32854511

[88] Fletcher-Watson S, Adams J, Brook K, et al. Making the future together: Shaping autism research through meaningful participation. Autism. 2018;23(4):943–953. 10.1177/1362361318786721 30095277 PMC6512245

[89] Jivraj J, Sacrey LA, Newton A, et al. Assessing the influence of researcher-partner involvement on the process and outcomes of participatory research in autism spectrum disorder and neurodevelopmental disorders: A scoping review. Autism. 2014;18(7):782–793. 10.1177/1362361314539858 24989447

[90] Pickard H, Pellicano E, den Houting J, Crane L. Participatory autism research: Early career and established researchers' views and experiences. Autism. 2022;26(1):75–87. 10.1177/13623613211019594 34088215 PMC8750139

[91] Crane L, Adams F, Harper G, Welch J, Pellicano E. Know your normal toolkit: What's your normal*?* Ambitious about autism. https://www.ambitiousaboutautism.org.uk/sites/default/files/youth-participation/toolkit/Ambitious-about-Autism-know-your-normal-toolkit.pdf Accessed December 16, 2022.

[92] Heasman B, Morrison K, Sasson N, Fletcher-Watson S, Crompton C, Milton D. Double empathy podcast: Ep. 1 part 1. YouTube. https://www.youtube.com/watch?v=TY1FBvJpzW8&t=7s Accessed December 16, 2022.

[93] Whitehouse A. 60 Second science—Loneliness and autism. YouTube. https://www.youtube.com/watch?v=zalJa0PnNgU&list=PLPYSm4ZweBNlDkT11XyNo3V13ceFkQJSu&index=12 Accessed December 8, 2022.

[94] Kapp SK, Gillespie-Lynch K, Sherman LE, Hutman T. Deficit, difference, or both? Autism and neurodiversity. Dev Psychol. 2013;49(1):59–71. 10.1037/a0028353 22545843

[95] Loomes R, Hull L, Mandy W. What is the male-to-female ratio in autism spectrum disorder? A systematic review and meta-analysis. J Am Acad Child Adolesc Psychiatry. 2017;56(6):466–474. 10.1016/j.jaac.2017.03.013 28545751

[96] Office for National Statistics. Ethnic group, England and Wales: Census 2021. https://www.ons.gov.uk/peoplepopulationandcommunity/culturalidentity/ethnicity/bulletins/ethnicgroupenglandandwales/census2021 Accessed December 16, 2022.

[97] Rafferty A. Ethnic penalties in graduate level over-education, unemployment and wages: Evidence from Britain. Work Employ Soc. 2012;26(6):987–1006. 10.1177/0950017012458021

[98] Merrells J, Buchanan A, Waters R. “We feel left out”: Experiences of social inclusion from the perspective of young adults with intellectual disability. J Intellect Dev Disabil. 2019;44(1):1–10. 10.3109/13668250.2017.1310822

