# Supplementary material for: Autistic Adults' Priorities for Future Autism Employment Research: Perspectives from the United Kingdom
Source: Autism Adulthood. 2024 Feb 28;6(1):72–85. doi: 10.1089/aut.2022.0087 (PMC10902279; doi:10.1089/aut.2022.0087)
Supplement: Supplemental data [file Supp_AppS1.docx]

**Appendix SA1. Example Research Questions**

| **Theme** | **Subtheme** | **Example Research Questions** |
| --- | --- | --- |
| 1. **Accessing employment** | 1.1 Opportunities for suitable employment | Which jobs might be particularly suitable for autistic people? |
|  |  | Which fields of employment might autistic people thrive in the most? |
|  |  | Which employers are actively seeking out autistic talent? |
|  |  | How effective are school-employment transition support/job seeking support programmes? |
|  |  | Which are the most effective school-employment transition support/job seeking support programmes? |
|  | 1.2 Autism-Employment Research Must Translate into Systemic Change | How can legislation ensure autistic people have equal access to employment? |
|  |  | What systemic changes are needed to ensure autistic people have equal access to employment? |
|  | 1.3 Exploring Challenges Surrounding Diagnosis and Disclosure | What are autistic people’s experiences of disclosing their diagnosis in the workplace? |
|  |  | What are the consequences of disclosing an autism diagnosis in the workplace? |
|  |  | How can we support autistic people to feel comfortable disclosing their diagnosis at work? |
|  | 1.4 The Impact of Intersectional Identities on Employment Experiences | What are the workplace experiences of autistic women? |
|  |  | What are the workplace experiences of autistic people from the LGBTQ+ community? |
|  |  | What are the workplace experiences of autistic people from minority ethnic backgrounds? |
|  |  | What are the workplace experiences of autistic people from a working-class background? |
|  | 1.5 A More Inclusive Recruitment Process | How can recruitment processes be improved? |
|  |  | Which recruitment processes are most favoured by autistic candidates? |
| 1. **Organisational culture** | 2.1 Exploring Stigma, Discrimination and Exploitation in the Workplace | To what extent do autistic people face stigma and discrimination in the workplace? |
|  |  | What is the relationship between experiences of workplace stigma and unemployment in the autistic population? |
|  |  | How well do workplace training programmes work to reduce autism-stigma and discrimination? |
|  |  | Which workplace training programmes are most effective in reducing autism-stigma and discrimination? |
|  | 2.2 Improving Understanding and Acceptance from Others | How effective are workplace training programmes at improving knowledge about autism? |
|  |  | How effective are workplace training programmes at improving autism acceptance? |
|  |  | How effective are workplace training programmes at improving attitudes toward autistic people? |
|  |  | Which workplace training programmes are most effective at improving colleagues understanding and acceptance of autism? |
|  |  | Do workplace training programmes about autism improve autistic employee experience? |
|  |  | How well do training programmes for autistic people (e.g., social-skills training) work to support autistic people in navigating the workplace? |
|  |  | What are the strengths of autistic employees? |
|  | 2.3 Better Networks of Support | How can autistic professionals be better supported at work? |
|  |  | How well does job coaching work to support autistic people in the workplace? |
|  |  | How well does mentoring work to support autistic people in the workplace? |
|  | 2.4 Access to Workplace Adjustments | What adjustments may be particularly beneficial for autistic people? |
|  |  | What are the productivity benefits of implementing workplace adjustments for autistic people? |
|  |  | How can employers be encouraged to implement workplace adjustments? |
| 1. **The Employment Journey** | 3.1 Experiences of Career Progression | What does successful work look like for autistic people? |
|  |  | What do autistic peoples’ work histories look like? |
|  |  | Do autistic people progress at the same rate as non-autistic people? |
|  |  | Do autistic people get paid fairly? |
|  |  | How can autistic people be better supported to progress in their career? |
|  | 3.2 Transitions Out of Employment | Why do autistic people leave employment? |
|  |  | What are autistic people’s experiences of retirement? |
|  |  | How can autistic people be supported in preparing for retirement? |
| 1. **The Negative Impact of Non-Inclusive Workplace Environments** | 4.1 Mental-Health Related Consequences of Employment | What are the mental-health consequences of employment for autistic people? |
|  |  | How can workplaces prevent poor mental-health outcomes for autistic people? |
|  | 4.2 Workplace Masking as a Perceived Necessity | What is the relationship between workplace masking and employment outcomes? |
|  |  | How can autistic people be better supported so they do not need to mask at work? |
|  |  | What are the potential benefits of workplace masking? |
